# Influence of Co-morbidities During SARS-CoV-2 Infection in an Indian Population

**DOI:** 10.3389/fmed.2022.962101

**Published:** 2022-08-01

**Authors:** Adrian Matysek, Aneta Studnicka, Wade Menpes Smith, Michał Hutny, Paweł Gajewski, Krzysztof J. Filipiak, Jorming Goh, Guang Yang

**Affiliations:** ^1^Immunidex Ltd., London, United Kingdom; ^2^Cognescence Ltd., London, United Kingdom; ^3^Clinical Analysis Laboratory, Silesian Centre for Heart Diseases, Zabrze, Poland; ^4^Faculty of Medical Sciences in Katowice, Students’ Scientific Society, Medical University of Silesia, Katowice, Poland; ^5^AGH University of Science and Technology, Krakow, Poland; ^6^Maria Skłodowska-Curie Medical Academy, Warsaw, Poland; ^7^Healthy Longevity Translational Research Program, Yong Loo Lin School of Medicine, National University of Singapore, Singapore, Singapore; ^8^Department of Physiology, Yong Loo Lin School of Medicine, National University of Singapore, Singapore, Singapore; ^9^National University Health System (NUHS), Centre for Healthy Longevity, Singapore, Singapore; ^10^Cardiovascular Research Centre, Royal Brompton Hospital, London, United Kingdom; ^11^National Heart and Lung Institute, Imperial College London, London, United Kingdom

**Keywords:** SARS-CoV-2, blood biomarkers, COVID-19, machine learning, vitamin D

## Abstract

**Background:**

Since the outbreak of COVID-19 pandemic the interindividual variability in the course of the disease has been reported, indicating a wide range of factors influencing it. Factors which were the most often associated with increased COVID-19 severity include higher age, obesity and diabetes. The influence of cytokine storm is complex, reflecting the complexity of the immunological processes triggered by SARS-CoV-2 infection. A modern challenge such as a worldwide pandemic requires modern solutions, which in this case is harnessing the machine learning for the purpose of analysing the differences in the clinical properties of the populations affected by the disease, followed by grading its significance, consequently leading to creation of tool applicable for assessing the individual risk of SARS-CoV-2 infection.

**Methods:**

Biochemical and morphological parameters values of 5,000 patients (Curisin Healthcare (India) were gathered and used for calculation of eGFR, SII index and N/L ratio. Spearman’s rank correlation coefficient formula was used for assessment of correlations between each of the features in the population and the presence of the SARS-CoV-2 infection. Feature importance was evaluated by fitting a Random Forest machine learning model to the data and examining their predictive value. Its accuracy was measured as the F1 Score.

**Results:**

The parameters which showed the highest correlation coefficient were age, random serum glucose, serum urea, gender and serum cholesterol, whereas the highest inverse correlation coefficient was assessed for alanine transaminase, red blood cells count and serum creatinine. The accuracy of created model for differentiating positive from negative SARS-CoV-2 cases was 97%. Features of highest importance were age, alanine transaminase, random serum glucose and red blood cells count.

**Conclusion:**

The current analysis indicates a number of parameters available for a routine screening in clinical setting. It also presents a tool created on the basis of these parameters, useful for assessing the individual risk of developing COVID-19 in patients. The limitation of the study is the demographic specificity of the studied population, which might restrict its general applicability.

## Introduction

The severe acute respiratory syndrome coronavirus type 2 (SARS-CoV-2) that causes the coronavirus disease 2019 (COVID-19) is a serious threat to human health and life. Due to the ease of spread and mutation of the virus, the World Health Organization (WHO) has declared COVID-19 a pandemic. Viral infection may be asymptomatic or symptomatic with varying degrees of severity. In some cases, SARS-CoV-2 infection leads to death of the patient (1).

The course of the SARS-CoV-2 infection may be influenced by several factors, including the presence of comorbidities in patients. Comorbidities, such as type 2 diabetes, affect the host immune response, which may be associated with a severe course of SARS-CoV-2 infection. In the context of type 2 diabetes, there is an increased release of pro-inflammatory cytokines, which can lead to cytokine storms in SARS-CoV-2 (+) patients. The occurrence of a cytokine storm correlates with a worse course of infection (1–4). Moreover, some changes in blood parameters have been observed in patients with SARS-CoV-2 (+), which may additionally affect the severity of the infection. Changes for example in the values of liver and kidney parameters, morphology and inflammatory markers in SARS-CoV-2 (+) patients may indicate a complex mechanism of infection and its long-term consequences (5).

Considering the wide disparity in the course of SARS-CoV-2 infection in patients and the need to define an effective therapy, the primary aim of our study was to define the physiological characteristics in patients infected with the SARS-CoV-2 virus, while presenting with concomitant diseases. The secondary aim of our study was to elucidate the underlying mechanisms that influence multiorgan dysfunction in COVD-19 infection. The contributing physiological parameters were ranked in terms of their relevance to the power of predictive model using machine learning.

## Materials and Methods

### Dataset

The analysed dataset consisted of blood test results from 5,000 patients from a digital healthcare system Curisin Healthcare (India). Two thousand five hundred of them were from patients (female, *n* = 1667; 67% and male, *n* = 833; 33%, age 25 – 78 years, mean = 51.6 years, median = 51.0 years) hospitalised due to infection with severe acute respiratory syndrome coronavirus 2 (SARS-CoV-2); the other two thousand and five hundred blood test results belonged to the control group (female, *n* = 755; 30%, male, *n* = 1,745; 70%; age 20 – 55, mean = 30.5 years, median = 30.0 years) and were collected before the coronavirus pandemic outbreak (2016-2018).

In blood biomarkers and parameters were excluded ones that are known to correlate with inflammatory responses such as C-reactive protein (CRP). Biomarkers that were not present in both datasets were also ruled out. The resulting set of data features enrolled in this study consisted of anthropometric parameters: age, gender; biochemical serum parameters: random glucose (RG), urea, alanine transaminase (ALT), cholesterol, creatinine, vitamin D; morphological parameters: red blood cell count (RBC Count); and functional parameters: estimated glomerular filtration rate (eGFR).

Glomerular filtration rate was not directly measured in the blood samples, instead the eGFR score was estimated using patient’s age, gender and serum creatinine level according to the simplified modification of diet in renal disease (MDRD) formula, described by the National Kidney Foundation (6), listed in Equation 1. below.

**Equation 1.** GFR definition.


GFR={C-1.154⁢x⁢ 186.3⁢x⁢A-0.203⁢x⁢ 0.742,G=F⁢e⁢m⁢a⁢l⁢eC-1.154⁢x⁢ 186.3⁢x⁢A-0.203,G=M⁢a⁢l⁢e

C, creatinine; A, age; G, gender.

The reference ranges for the analysed parameters are listed below in Table 1.

**TABLE 1:** Reference ranges of the analysed parameters.

Property	Reference Range
RG	74 – 100 mg/dl
Serum Urea	13 – 40 mg/dl
RBC Count	3.8 – 4.8 million/cumm
ALT	≤ 34 U/L
Cholesterol	≤ 200 mg/dl
Creatinine	0.6 – 1.1 mg/dl
Vitamin D	30 – 100 ng/dl

*ALT, alanine transaminase; RBC Count, red blood cells count; RG, random serum glucose.*

### Correlations

The correlations between each of the features in the population and the presence of the SARS-CoV-2 infection were calculated using Spearman’s method (see **Equation 3**).

**Equation 2.** Pearson’s correlation.


for⁢x∈X,y∈Y

$$\rho \,\left(\text{x},y\right)=\,\frac{\sum \left[\left({\text{x}}_{\text{i}}-\bar{\text{x}}\right)\,\left({\text{y}}_{\text{i}}-\bar{\text{y}}\right)\right]}{\sigma \,x*\sigma \,y}$$

$\bar{x}$, mean of X; $\bar{y}$, mean of Y; σx, standard deviation of X; σy, standard deviation of Y.

**Equation 3.** Spearman’s correlation.


for⁢x∈X,y∈Y

$${\text{r}}_{\text{s}}\,\left(\text{x},y\right)=\rho_{\text{R}\,\left(\text{X}\right),\text{R}\,\left(\text{Y}\right)}=\,\frac{\text{cov}\,\left(\text{R}\,\left(\text{X}\right),R\,\left(\text{Y}\right)\right)}{\sigma_{\text{R}\,\left(\text{X}\right)}\,\sigma_{\text{R}\,\left(\text{Y}\right)}}$$

ρ, Pearson’s correlation (**Equation 2**) applied to rank variables; cov (R(X), R(Y)), covariance of rank variables; σ_*R(X)*_, standard deviation of rank variable of X; σ_(R(Y)_, standard deviation of rank variable of Y.

### Feature Importance

To discover how each feature affects the possibility of severe SARS-CoV-2 infection, the importance of each feature was evaluated by fitting a Random Forest (7) machine learning model to the data and examining which features had the highest predictive value. The accuracy measure of the predictive model was the F1 Score as defined by **Equation 4**. Results of this analysis were presented graphically in section “Results” in Figure 2.

**FIGURE 1:**
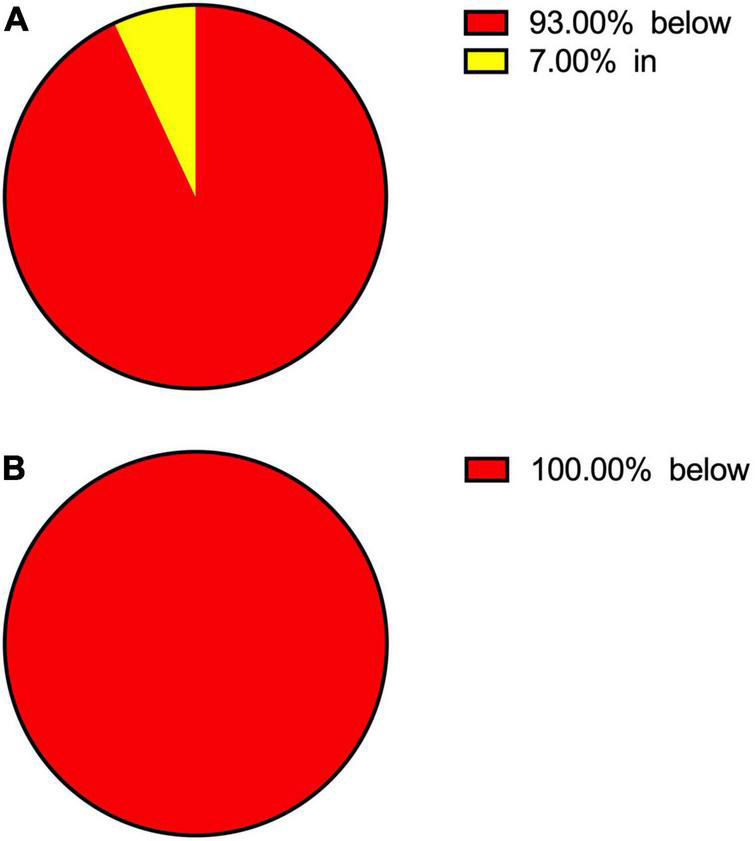
Patients’ vitamin D status in: **(A)** SARS-CoV-2 (–) group; **(B)** SARS-CoV-2 (+) group.

**FIGURE 2:**
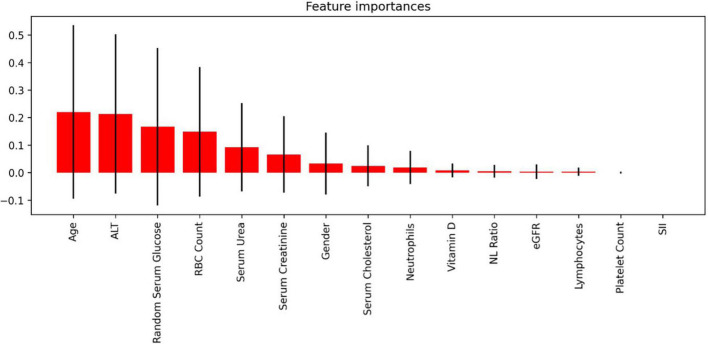
Feature importance of each parameter assessed for the predictive model.

**Equation 4.** F1 Score measuring the accuracy of the predictive model.


$${\text{F}}_{1}=\frac{\text{tp}}{\text{tp}+\frac{1}{2}\,\left(\text{fp}+\text{fn}\right)}$$

tp, number of true positives; fp, number of false positives; fn, number of false negatives.

### Vitamin D in Relation to NL Ratio and SII

Additional analysis of the possible correlation between the concentration of vitamin D in SARS-CoV-2 (+) patients and the values of parameters such as Neutrophil-Lymphocyte Ratio (NL Ratio) and Systemic immune-inflammation index (SII) was conducted in our study. For the purpose of the analysis the patients were divided into tertiles in terms of Vitamin D level: I tertial (I T): Vit D (1.11 - 9.40], II tertial (II T): Vit D (9.40 - 17.69], III tertial (III T): Vit D (17.69 - 25.97]; followed by assessment of the NL Ratio values and of the systemic immune inflammation index, which was calculated as defined in **Equation 5**.

**Equation 5.** Calculation of systemic immune inflammation index.


$$\text{SII}=\frac{P\,x\,N}{\text{L}}$$

P, N, and L represent platelet, neutrophil and lymphocyte counts in cells/L.

## Results

Results of biochemical examinations performed on the material collected from both SARS-CoV-2 (+) and SARS-CoV-2 (−) groups are presented below in Table 2. as the number of patients with results falling into the respective groups created in relation to the reference range (below, above or in reference range).

**TABLE 2:** Patients’ outcomes in SARS-CoV-2 (–) (n = 2,500) and SARS-CoV-2 (+) group (*n* = 2,500).

Property	Below reference range N;%		Above reference range N;%		Within reference range N;%	
	(–)	(+)	(–)	(+)	(–)	(+)
Random Glucose	286; 11	5; ∼0	780; 31	2162; 86	1434; 57	333; 13
Blood Urea	995; 40	0; 0	0; 0	88; 4	1505; 60	2412; 96
RBC Count	34; 1	651; 26	1707; 68	560; 22	759; 30	1289; 52
ALT	–	–	1384; 55	0; 0	1116; 45	2500; 100
Cholesterol	–	–	799; 32	1414; 57	1701; 68	1086; 43
Creatinine	123; 5	0; 0	541; 21	0; 0	1836; 74	2500; 100
Vitamin D	2324; 93	2500; 100	3; ∼0	0; 0	173; 7	0; 0

*ALT, alanine transaminase; RBC Count, red blood cells count.*

The percentage of patients in each reference-range-related group in terms of serum vitamin D levels is presented in form of a circle chart for both infection-related groups of patients in Figure 1.

To allow a more profound insight into the distribution of parameters values, the number of patients in relation to the exact values of parameters are presented as the histograms for each respective parameter in the Supplementary Materials in Supplementary Figure 1.

The correlations between the studied parameters and SARS-CoV-2 infection in patients were calculated as described in Section “Materials and Methods.” It must be noted that due to simultaneous influences of each parameter, none of them may offer sufficient reliability in terms of prediction of SARS-CoV-2 infection. Results of correlations analysis are listed below in Table 3.

**TABLE 3:** Correlations between features and SARS-CoV-2 features.

Property	Correlation with SARS-CoV-2
Age	0.671854
Gender	0.364978
RG	0.652085
Serum Urea	0.551667
RBC Count	–0.591819
ALT	–0.662445
Serum Cholesterol	0.255992
Serum Creatinine	–0.368583
Vitamin D	–0.148394
eGFR	–0.067112
Neutrophils	0.229344
Lymphocytes	–0.087234
NL Ratio	0.138331
Platelet Count	0.020377
SII	0.103531

*ALT, alanine transaminase; eGFR, estimated glomerular filtration rate; NL Ratio, Neutrophil-lymphocyte ratio; RBC Count, red blood cells count; RG, random serum glucose; SII, Systemic immune-inflammation index.*

To further examine the influence of respective features on infection severity in relation to the complexity of simultaneous influences of studied features, the predictive model was created as described in Section “Feature Importance” and presented below in Figure 2. The model itself was a successful one, with an accuracy of 97% in differentiating positive from negative cases of SARS-CoV-2 infection.

**FIGURE 3:**
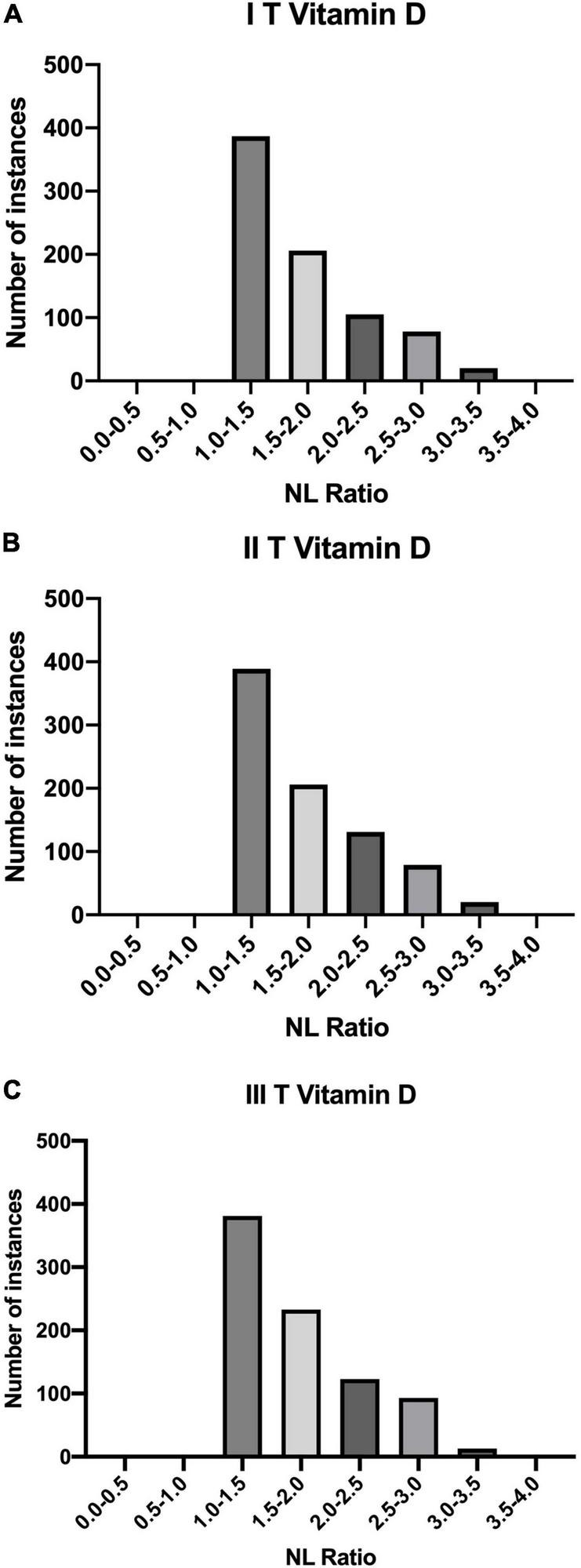
Quantities of patients in buckets of NL Ratio values, grouped according to their Vitamin D status: **(A)** I T (1.11 – 9.40]; **(B)** II T (9.40 – 17.69]; **(C)** III T (17.69 – 25.97].

The results of analysis of NL Ratio values structure in relation to the vitamin D levels are presented, respectively, below in Table 4 and Figure 3, whereas relation between SII and vitamin D levels in patients is summarised in Table 5.

**TABLE 4:** Quantities of patients divided according to the Vitamin D levels in terms of NL Ratio.

NL Ratio	Patients Count		
	I T (1.11 – 9.40]	II T (9.40 – 17.69]	III T (17.69 – 25.97]
0.0 - 0.5	0	0	0
0.5 - 1.0	0	0	0
1.0 - 1.5	387	389	381
1.5 - 2.0	206	206	233
2.0 - 2.5	105	131	123
2.5 - 3.0	78	79	93
3.0 - 3.5	20	20	13
3.5 - 4.0	0	0	2

*NL Ratio; neutrophil-lymphocyte ratio.*

**TABLE 5:** The number of patients divided according to the Vitamin D levels in terms of SII.

SII	Patients Count		
	I T (1.11 – 9.40]	II T (9.40 – 17.69]	III T (17.69 – 25.97]
Low	315	315	321
High	493	519	535

*SII, systemic immune inflammation index.*

## Discussion

The collected data indicate that the course of SARS-CoV-2 infection may depend on numerous factors. In the present study, the influence of gender, age and changes in blood parameters values of Indian patients were addressed and examined.

### Age

A strong correlation between age and severe course of SARS-CoV-2 (0.671007) was observed, which suggests that the risk of developing severe SARS-CoV-2 increases with age. This observation was already reported in the early meta-analyses concerning risk factors for mortality in the course of COVID-19 infection (8). A similar association was also found in study (9) conducted on a group of patients from the New York City metropolitan area and in another study involving data from two Wuhan hospitals – results of these studies showed that age correlates with the need for hospitalization and severity of SARS-CoV-2 infection (10). This dependence is determined by several factors that characterize the ageing process - the percentage of diagnosed comorbidities in those patients, i.e., diabetes and cardiovascular diseases, increases with age. Ageing is also naturally associated with the decreasing functioning of the immune system, which in turn leads to changes in adaptive and innate immunity (11, 12).

### Gender

In the presented analysis of the influence of the patient’s gender during SARS-CoV-2, a significant correlation between adverse prognosis and female gender was observed, contrary to the results of a previous report (13) that reported higher mortality among men (8). The differences in the course of SARS-CoV-2 infection among women and men may result from the influence of social and biological factors in the latter, e.g., higher expression of angiotensin-converting enzyme 2 (ACE2) found in male patients, which may facilitate entry of the virus into the host cell (14, 15). Due to the global spread of the COVID-19, the differences in societies-specific characteristics in terms of gender may additionally hinder the analysis of this factor. As case fatality ratio for males is higher in COVID-19 than for females in global data analysis, it is worthy further research (16).

**FIGURE 4:**
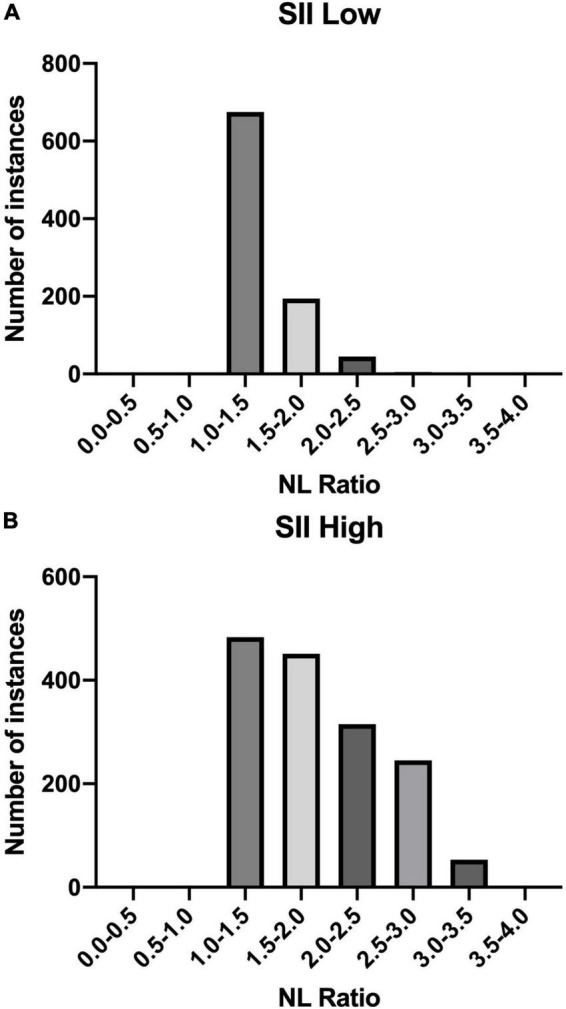
NL Ratio values structure in the studied population. The patients were grouped according to the SII index: **(A)** Low SII; **(B)** High SII.

The distribution of NL Ratio values in patients grouped according to their SII index (high: SII < 410/low: SII ≥ 410) is presented below in Figure 4.

### Biochemical Parameters

#### Random Blood Glucose

The results of RG examination in recruited patients indicate that hyperglycaemia correlates with a severe course of SARS-CoV-2. This finding was already reported in an Italian study (17). The random blood sugar level was elevated in over 30% of patients in the control group and over 86% of patients in the SARS-CoV-2 (+) group. Another study also reported that 51.5 and 57.4% of severely and critically ill patients were diagnosed with diabetes (18). High glucose concentration directly impacts the course of the infection, and also through the development of further complications, such as diabetic ketoacidosis and vascular comorbidities (atherosclerosis, peripheral arteriosclerosis) it influences this process indirectly (19).

#### Vitamin D

The association of vitamin D concentrations and the course of SARS-CoV-2 was assessed due to the high importance of Vitamin D in the activation of immune defence. Vitamin D status was below the reference range threshold in almost all patients in the control group and all SARS-CoV-2 (+) group patients (see Figure 1). This could be a possible explanation for a relatively low correlation parameter between SARS-CoV-2 infection and Vitamin D in the presented results. Other studies have shown that groups of patients with severe course of SARS-CoV-2, requiring hospitalization in the ICU and showing higher mortality, have decreased serum concentrations of Vitamin D (20, 21).

There is a significant influence of changes in both blood sugar and Vitamin D status in the immune system (22, 23). Insulin resistance and type 2 diabetes mellitus are two conditions associated with a pro-inflammatory status. Consequently, elevated systemic concentrations of inflammatory markers, including cytokines, are observed in the blood, which in turn correlates with a higher probability of a cytokine storm (2, 4). Also, normal concentrations of vitamin D reduce the risk of a cytokine storm by reducing the systemic concentration of pro-inflammatory cytokines such as IL-6 and TNF-α. The significantly higher concentration of pro-inflammatory cytokines in circulation correlates with the severe course of SARS-CoV-2 (24, 25). Nonetheless, results similar to the presented data in terms of differences in vitamin D status between COVID-19 patients and population-based controls were reported, suggesting that despite the proven potential causality in interactions of vitamin D levels and COVID-19 pathogenesis, the alterations of vitamin D status do not influence the severity of disease course (26). On the other hand, there are some meta-analyses showing that vitamin D supplementation in SARS-CoV-2 positive patients has the potential to positive impact their outcomes (27).

NL Ratio is regarded as a prognostic marker of systemic inflammation. It is an important element in the diagnosis of SARS-CoV-2(+) patients. Some studies indicate that its value correlates with the severe course of the disease and mortality (28). Apart from the assessment of inflammation, the NL Ratio is also used to analyse the course of other diseases, including cardiovascular diseases and cancer. Due to its numerous benefits, this parameter is assessed more frequently during SARS-CoV-2, compared to other inflammatory parameters. Its value can be assessed on the basis of one of the basic tests, i.e., morphology, taking into account the values of relative neutrophils and lymphocytes count. NL Ratio is characterized by a higher specificity and sensitivity compared to blood cell count. Moreover, its value increases faster, which is useful in the early assessment of the patient’s condition. Other studies shows that a higher value of NL Ratio correlates with a more severe course of SARS-CoV-2. It has been shown that higher NL Ratio values occur in people with a significant amount of comorbidities (29–31). In this study it was shown that among the tertiles of Vitamin D values, the biggest number of patients with SARS-CoV-2 (+) had an NL Ratio value of 1.0-1.5.

SII is another important predictive marker in SARS-CoV-2 (+) patients. It is used to assess the prognosis in people diagnosed with pulmonary embolism, obesity, sepsis, cancer and diseases accompanied by inflammation (32–36)., SII has been used as an indicator of severe course of SARS-CoV-2 (37, 38). In our study, each of the tertiles of vitamin D was correlated with SII high value (SII High: ≥ 410). In each of the tertiles of vitamin D, we included the division into low and high SII values. Our results showed that patients with high SII values predominated in each tertiles (SII High: ≥ 410).

Another analysis concerned the correlation between the SII and NL Ratio values. The highest percentage of patients (*n* = 675) in the SII-Low value group had an NL Ratio of 1.0 - 1.5. However, in the SII High group, the distribution of patients according to the NL Ratio value was as follows: the largest number of patients are in the NL Ratio ranges: 1.0 - 1.5 (*n* = 483) and 1.5 - 2.0 (*n* = 451). In SII-High values, the distribution of patients on NL Ratio values was more diverse.

Our results indicate that SII can be a reliable biomarker that is easily tested than those based on cytokines or coagulation system markers (39, 40).

#### Cholesterol

Our data confirmed the association between elevated cholesterol levels and the severity of course of SARS-CoV-2 infection. A study of a cohort of 3,988 critically ill Italian patients demonstrated that hypercholesterolaemia may be classified as a risk factor for increased mortality in patients SARS-CoV-2(+) admitted to the ICU (41). This observation may be associated with an increased risk of developing cardiovascular diseases in patients with hypercholesterolaemia, as well as with its influence on one of the components of lung surfactant, which is an important protection in the respiratory system (42–45).

### Morphologic Parameters

In an examination of blood morphology, a focus was put on the basic parameters, such as the number of RBC count, due to their accessibility in common clinical practice. The present results show that a low RBC count correlates in studied groups with a higher percentage of hospitalized patients and with a more severe course of infection. This observation may be related to the numerous important functions performed by RBC in the human body. Similar results were presented in a study performed in hospitals in Dhaka, Bangladesh, which indicated the likely impact of a reduction in red blood cell counts on the severity of SARS-CoV-2. The patients in the quoted study, who had a worse prognosis, were found to be present with decreased RBC count. This report also suggests a relationship between the number of RBCs and the concentration of haemoglobin (46). Nonetheless, the impact of changes in RBC Count over the course of SARS-CoV-2 infection requires more research.

### Renal and Hepatic Parameters

In the population of the present study, the patients hospitalized for SARS-CoV-2 were presented with changes in renal parameters such as serum urea, creatinine and GFR. The analysis discovered an increase in the level of serum urea and a decrease in the concentration of creatinine, which consequently influenced the GFR values. The strongest correlation among these parameters was observed for serum urea (0.555459). A study by Hachim et al. (47) showed that elevated urea levels correlated with a higher probability of patient admission to the ICU and a more severe course of the infection. Increased creatinine levels were also observed in this group of patients. The results presented by Chen et al. (13) agree with the assumption that patients developing kidney disorders during SARS-CoV-2 (+) are more likely to develop more severe complications in the course of the disease. Higher levels of both blood urea nitrogen and creatinine were described in patients who died of SARS-CoV-2 than in the recovered ones.

The impact of liver disease on the course of SARS-CoV-2 infection was also a scope of the presented study. The liver is an important organ for maintaining the homeostasis of the organism, therefore the disturbance of its function correlates with the occurrence of complications (48, 49). For this purpose, the changes in the level of standard liver marker ALT were examined among the studied groups. The analysis of the collected data shows that a decreased level of ALT is observed among the SARS-CoV-2 (+), contrary to results of a study performed by Chen et al. (13), which showed an increased concentration of this liver parameter in the deceased. The median (IQR) value in this study was for dead patients: ≤ 41 U/L: 23.0 (15.0-38.0); and for the recovered: > 41 U/L: 60.

Two crucial results differentiating the present study from others relate to our findings on the concentrations of ALT and creatinine. To reconcile those contradictory findings, the percentage of patients in study groups, both present and previous, with co-existing liver disease before developing SARS-CoV-2 needs to be determined. Another possible reason for such results could be the characteristic of the groups in terms of SARS-CoV-2 severity at the moment of taking blood tests. Patients enrolled in the Chen et al. (13) study were described as moderate to critically ill. 41.2% of the laboratory results presented in their study came from patients who eventually died, which could strongly influence the results. The present study recruited hospitalized patients, though the more detailed information on their clinical condition, exact severity of the disease and medical history concerning chronic diseases are lacking. Further research is required to assess the influence of liver and kidneys’ function on the severity of the COVID19. Therefore, whether acute liver disease and acute kidney injury during the course of SARS-CoV-2 could be developed at the more advanced and severe stadium as well as whether the potential distinction in ALT and creatinine outcomes in both studies could be influenced by the presence of chronic liver diseases before the studies remain unknown.

### Predictive Model

Mathematical models are valuable tools for the prevention and control of infectious diseases, including SARS-CoV-2. Thanks to them, it is possible to determine the relationship between various processes and to assess the dynamics of the disease spread. It also helps to establish a vaccination strategy. For this reason, during the SARS-CoV-2 pandemic, many researchers have proposed numerous mathematical models based on various correlations. The mathematical models took into account, *inter alia*, the method of transmission, the impact of quarantine and isolation, pharmaceutical and non-pharmaceutical interventions (50, 51). Most of the models developed during the SARS-CoV-2 pandemic were based on the Susceptible-infected-recovered (SIR) model and the Susceptible-Exposed-Infected-Removed (SEIR) model. These models are used to assess the spread of the virus (52). Researchers introduced numerous modifications to the classic SIR model due to the dynamics of SARS-CoV-2 spreading. Cooper et al. (53) adapted the SIR model to the increasing over time number of susceptible individuals. In the Susceptible-Exposed-Infected-Removed model, each person can be assigned to the following category susceptible (S), exposed (E), infected (I) and recovered/removed (R) at any time during the epidemic. The researchers also made modifications to the SEIR model. One of them concerns the impact of human migration on the spread of the virus (54). Other modifications include assessing the impact of using face masks (55).

The biochemical parameters were studied in terms of disease prediction and triage already in previous coronavirus outbreak – Middle East respiratory syndrome coronavirus (MERS-CoV). The results showed that the routine parameters may turn out to be useful in clinical decision making (56). Similarly, the basic blood biomarkers were a focus of research in early stages of COVID-19 pandemic, though the reports indicated a rather moderate efficiency of these parameters in differentiating infected patients from healthy ones (57, 58). More optimistic results concerned the estimation of mortality risk (59).

The recent advances in bioinformatics enable creation of complex models, which based on the patient’s data assess the risk of infection or severity of potentially acquired disease. By harnessing the power of machine learning and artificial intelligence, it is possible to offer an alternative to standard biochemical diagnostic tools, such as reverse transcription-polymerase chain reaction (RT-PCR) assays, which in some parts of the world remain expensive and difficult to obtain (60). It may also be useful in analysing a dynamic of current clinical state of patients, and on this basis nowcasting the further disease development (61).

Analysis of feature importance in the current study returned a valuable insight into the potential of studied parameters in terms of assessing the patient’s risk of SARS-CoV-2 infection severity, providing a satisfying prediction accuracy of 97%. The main features of the predictive model created in this study were basic parameters obtained during the regular blood test – RBC count, serum glucose, serum urea, as well as the anthropometric measure – age. These results expand the spectrum of features useful in the COVID-19 prediction, as the previous study harnessed the methods of deep learning and the Random Forest machine learning model reported the efficacy of other parameters: lactate, the absolute level of immature granulocytes, respiratory rate, haemoglobin, procalcitonin, hematocrite (62). The only matching parameter between this and previous studies was serum urea, which in both studies was recognized as an important factor. A model presented in mentioned above study showed lower precision (current study: 0.98 vs. Aktar et al.: 0.90) and recall (0.97 vs. 0.90), F1 score (0.98 vs. 0.90) accuracy (0.97 vs. 0.89) and AUC (0.99 vs. 0.89). Another earlier report also differed in terms of included biomarkers, as despite the similarities concerning included blood count parameters, the biochemical parameters set was different for previous and current study. Interestingly, in the cited study the blood count parameters such as neutrophil, basophil and eosinophil count had similar feature importance as age, which in the current study showed the highest importance. The most important features were lactate dehydrogenase and C-reactive protein, which were not included in the current study (63). It must be noted, however, that this study was conducted on smaller population (2,500 patients in current study vs. 1,455 cases in Goodman-Meza et al.) and led to creation of model characterised by lower AUC value (0.99 vs. 0.91) and area under precision recall curve (1.00 vs. 0.76). The above studies were all based on the biochemical parameters, nonetheless the created models could be enhanced by including an analysis X-ray or computer tomography images, as suggested by the previous reports (64, 65). Further research in this matter is necessary.

As shown with above results, the machine learning may be a key for assessing the SARS-CoV-2 infection risk without the necessity of applying the standard diagnostic measures. Other studies explored also the possibilities of estimating a mortality risk (66–68), as well as the severity of the COVID-19 course (69) based on the simple biochemical parameters. The cardiovascular component of COVID-19 may also be evaluated using machine learning models (70).

## Limitations

The difference in our analysis compared to other studies may be associated with a disproportionate number of women compared with men with SARS-CoV-2 who were enrolled in this study. The higher number of women may influence the observation that the female gender correlates with a more severe course of infection. The demographic variables of the studied population may also be a factor causing differences in the results regarding some of the parameters. Moreover, the analysed data is limited to the residents of India, which may also affect the obtained results. The influence of additional factors on the obtained results should also be taken into account.

The differences in presented ALT and creatinine levels compared to other studies may stem from the analysis of the different demographic groups included in the study. The influence of inter-individual variability, the treatment regimens used and the stage of the disease at which patients were admitted should also be taken into consideration.

Further research is necessary to adequately respond to the influence of limitations on the results and to evaluate the observations of the present study.

## Conclusion

Assessment of the impact of pre-existing comorbidities and changes in the biochemical and morphological parameters observed in SARS-CoV-2 patients in the course of the disease may contribute to a better understanding of the influence of each of these individual factors on the pathology. Therefore, it could consequently affect the selection of appropriate therapy and the reduction of possible complications. Presented results indicate the importance of adequate vitamin D supplementation and maintaining the physiological functions of the liver and kidneys for reducing the risk of severe COVID-19 course. Standard serum parameters, such as red blood cell count, serum glucose, urea, ALT, cholesterol and creatinine, are efficient in predicting the patient’s condition in terms of SARS-CoV-2 infection.

## Data Availability Statement

The raw data supporting the conclusions of this article will be made available by the authors, without undue reservation.

## Ethics Statement

Ethical review and approval was not required for the study on human participants in accordance with the local legislation and institutional requirements. Written informed consent was obtained from the individual(s) for the publication of any potentially identifiable images or data included in this article.

## Author Contributions

AM designed and coordinated the project. PG did the AI experiments. AM, AS, MH, and WS wrote the manuscript. KF, JG, and GY revised it critically for important intellectual content. All authors approved the version to be published.

## Conflict of Interest

AM and WS were employed by Cognescence Ltd., and Immunidex Ltd. The remaining authors declare that the research was conducted in the absence of any commercial or financial relationships that could be construed as a potential conflict of interest.

## Publisher’s Note

All claims expressed in this article are solely those of the authors and do not necessarily represent those of their affiliated organizations, or those of the publisher, the editors and the reviewers. Any product that may be evaluated in this article, or claim that may be made by its manufacturer, is not guaranteed or endorsed by the publisher.
