# Supplementary material for: Influence of Co-morbidities During SARS-CoV-2 Infection in an Indian Population
Source: Front Med (Lausanne). 2022 Aug 1;9:962101. doi: 10.3389/fmed.2022.962101 (PMC9377050; doi:10.3389/fmed.2022.962101)
Supplement: Supplementary file 1 [file Data_Sheet_1.docx]

Supplementary Material

## Supplementary Figures

**a)**


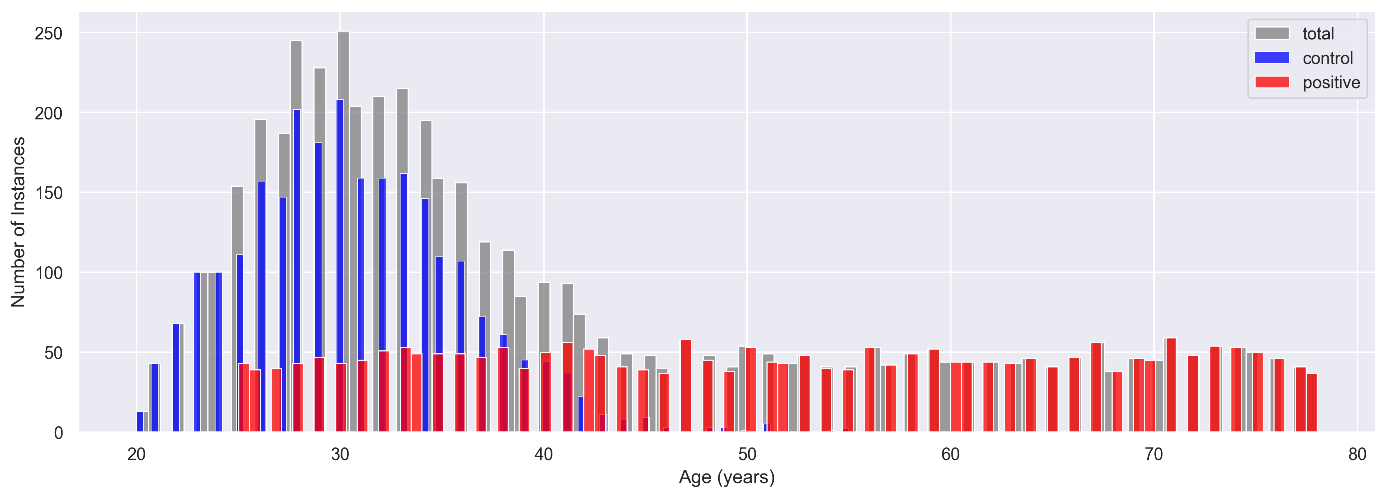


**b)**


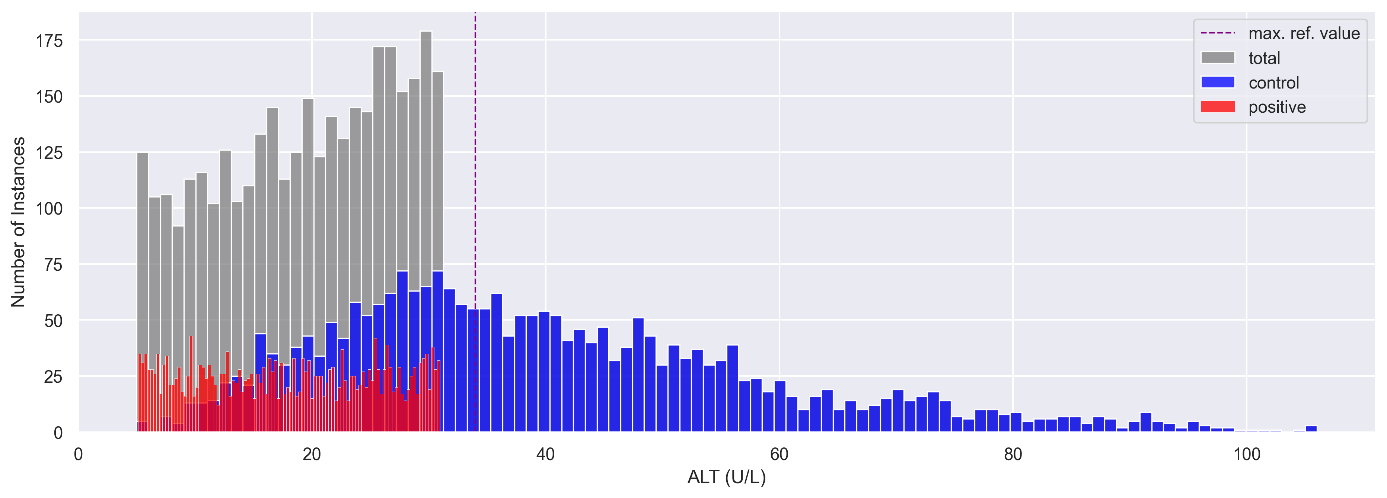


**c)**


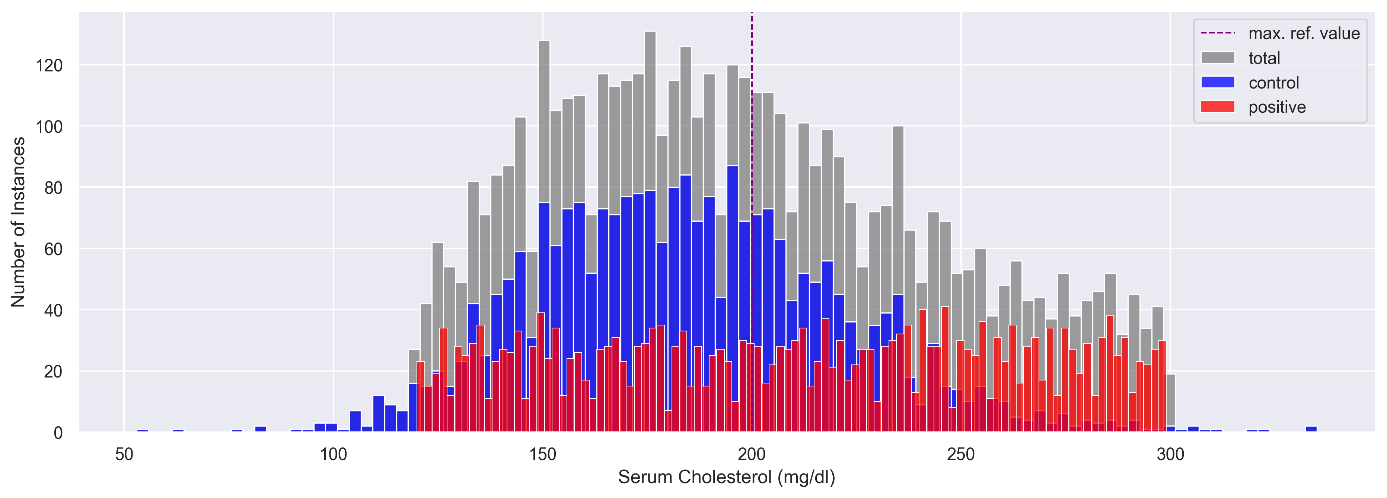


**d)**


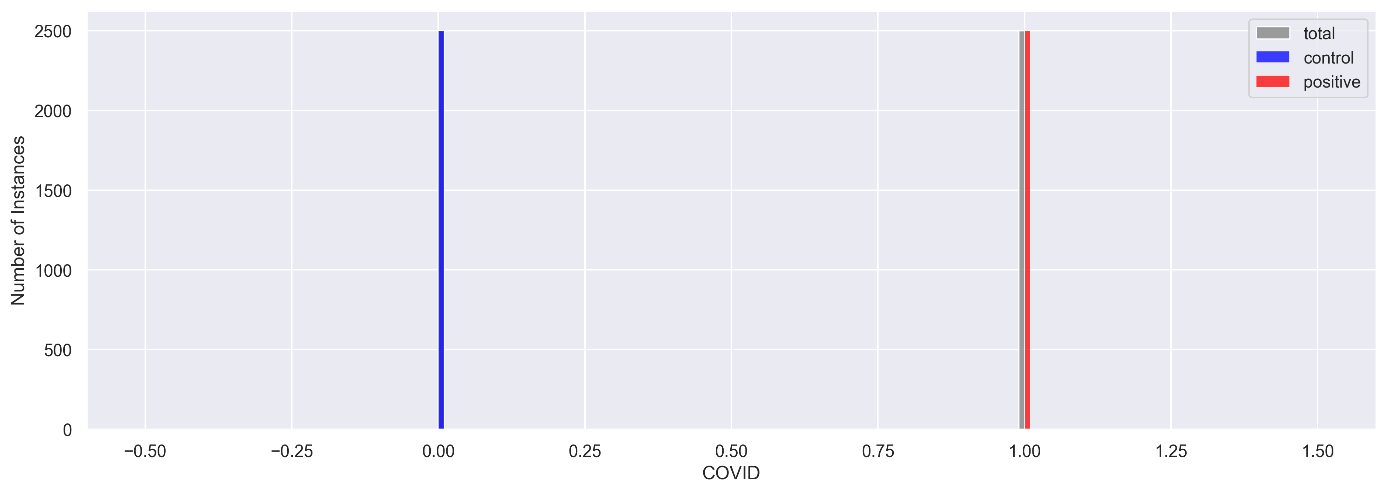


**e)**


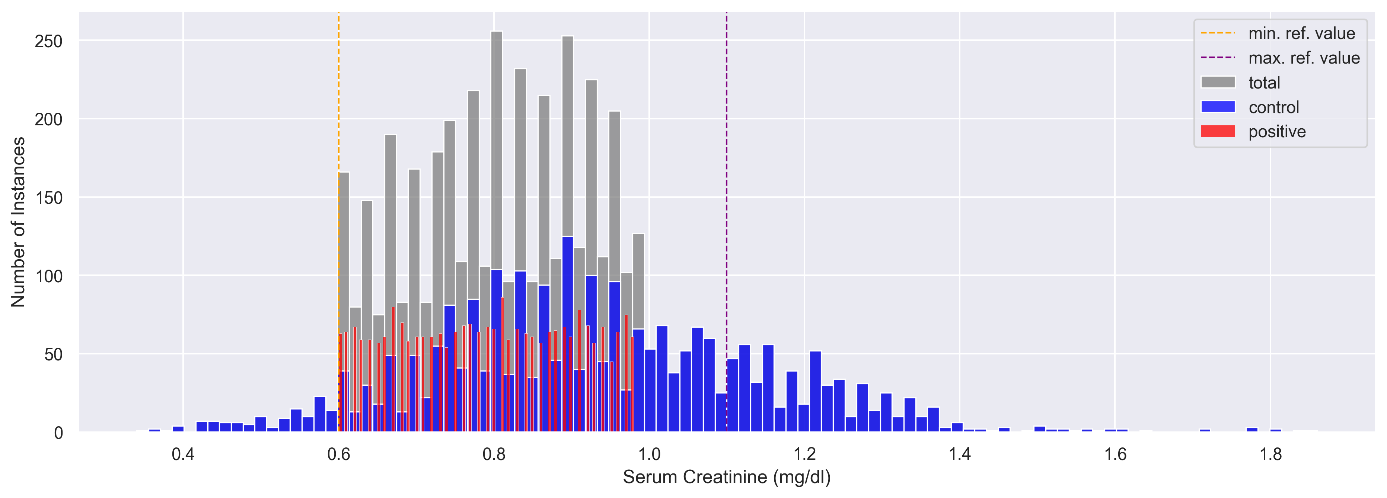


**f)**

**
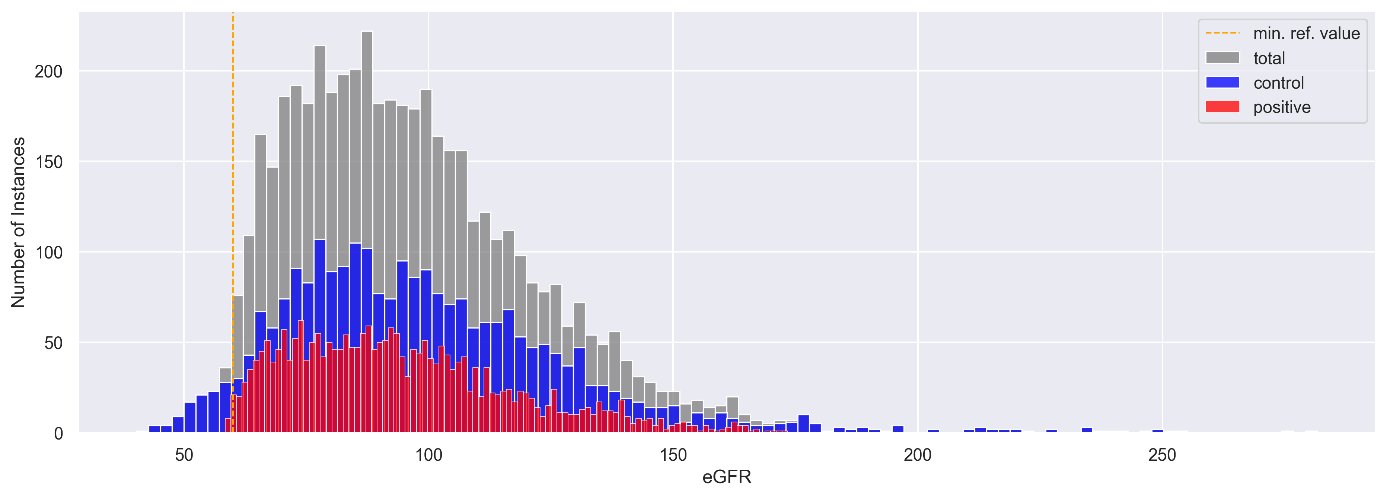
**

**g)**


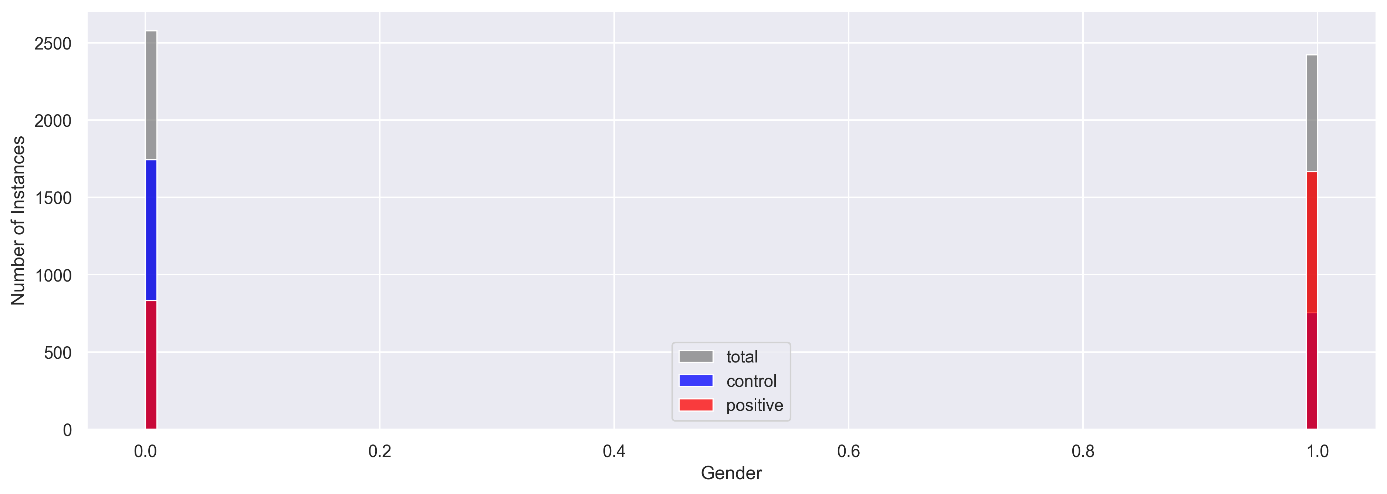


**h)**


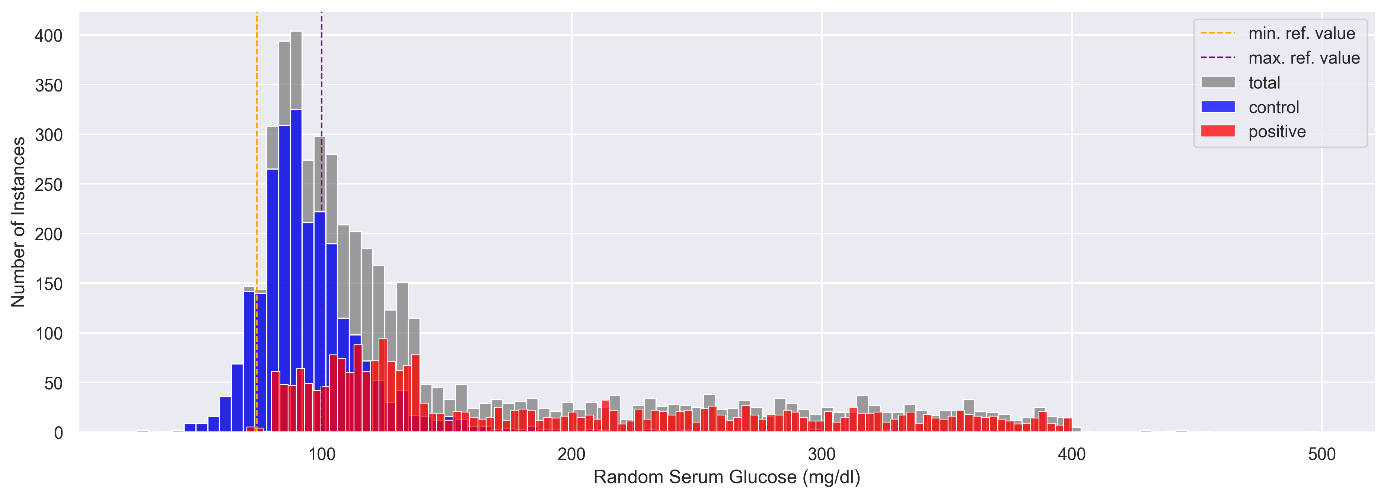


**i)**


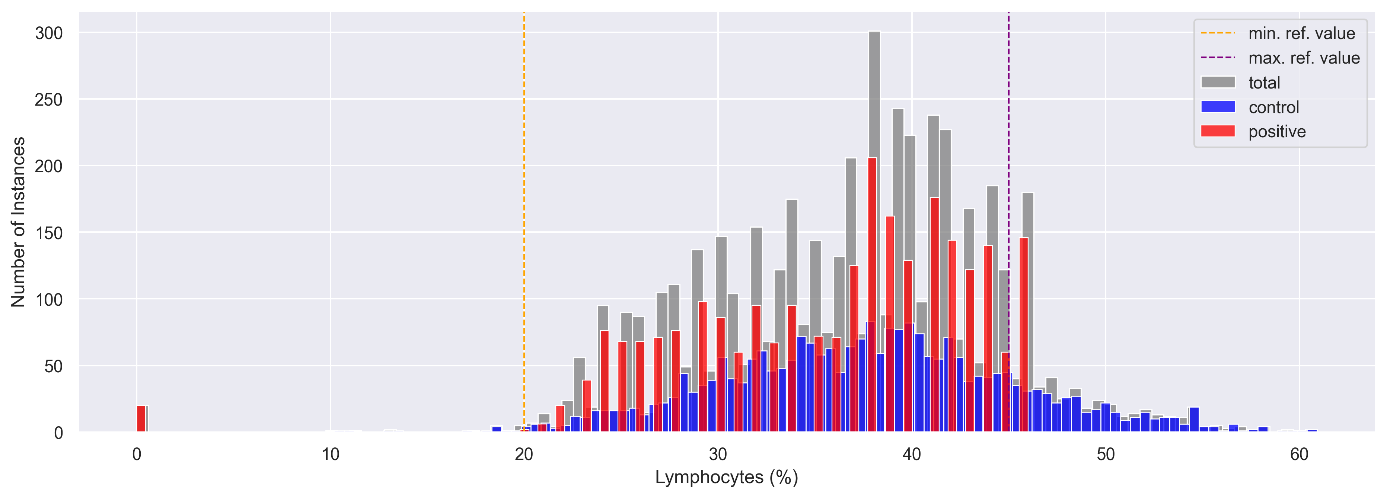


**j)**


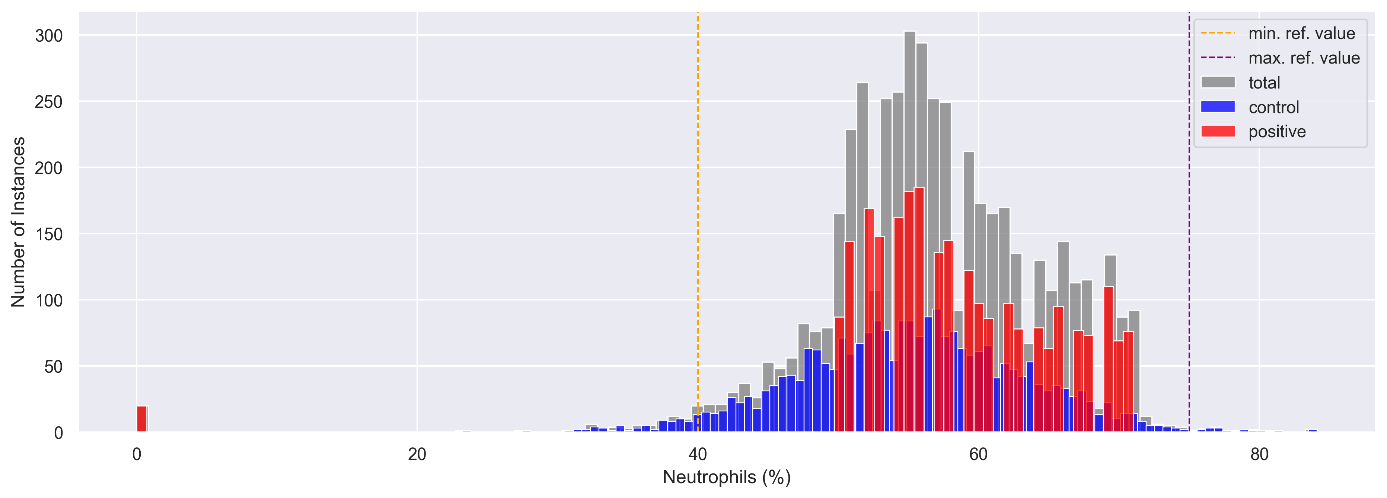


**k)**

**
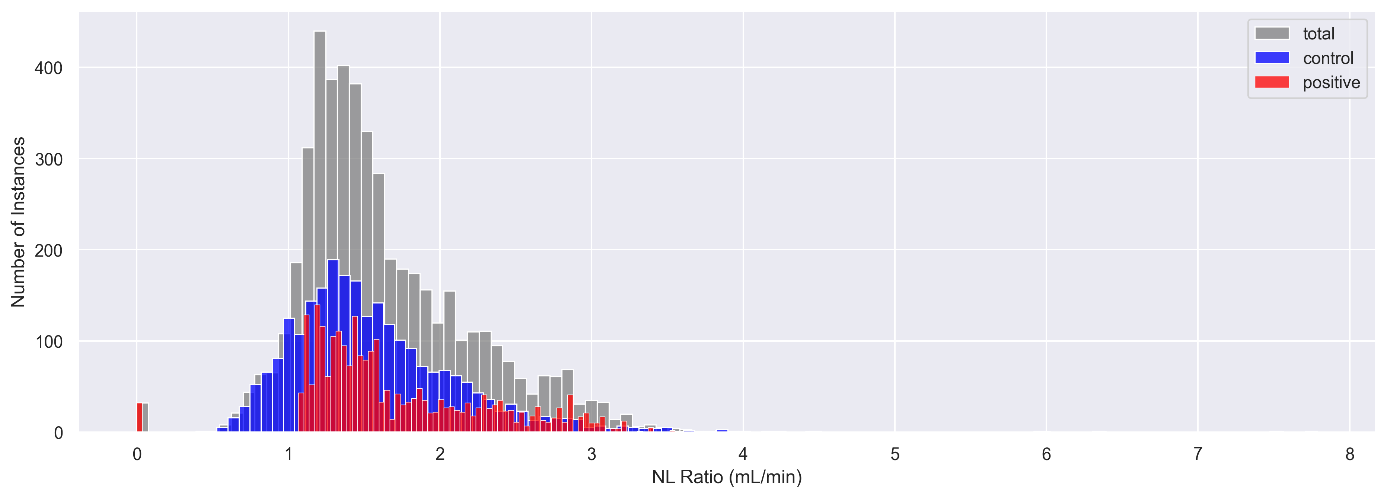
**

**l)**


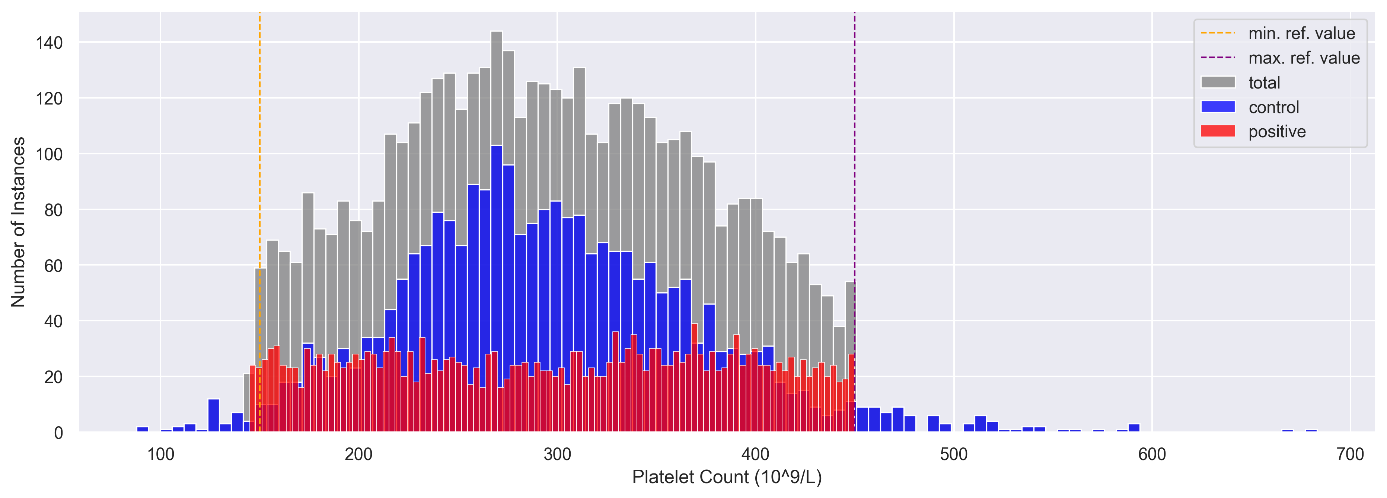


**m)**

**
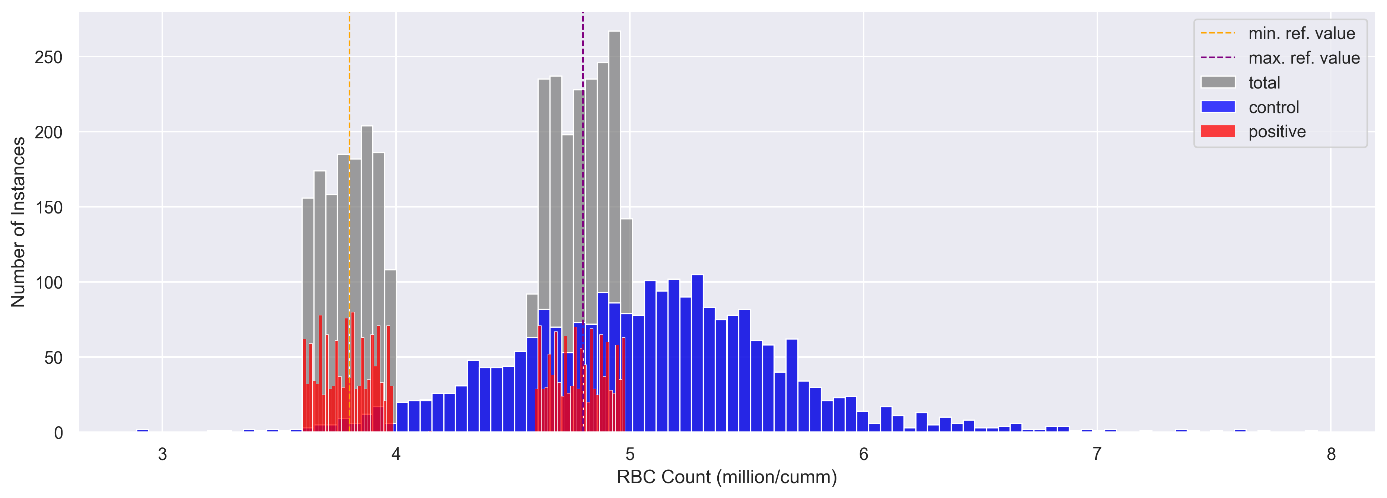
**

**n)**

**
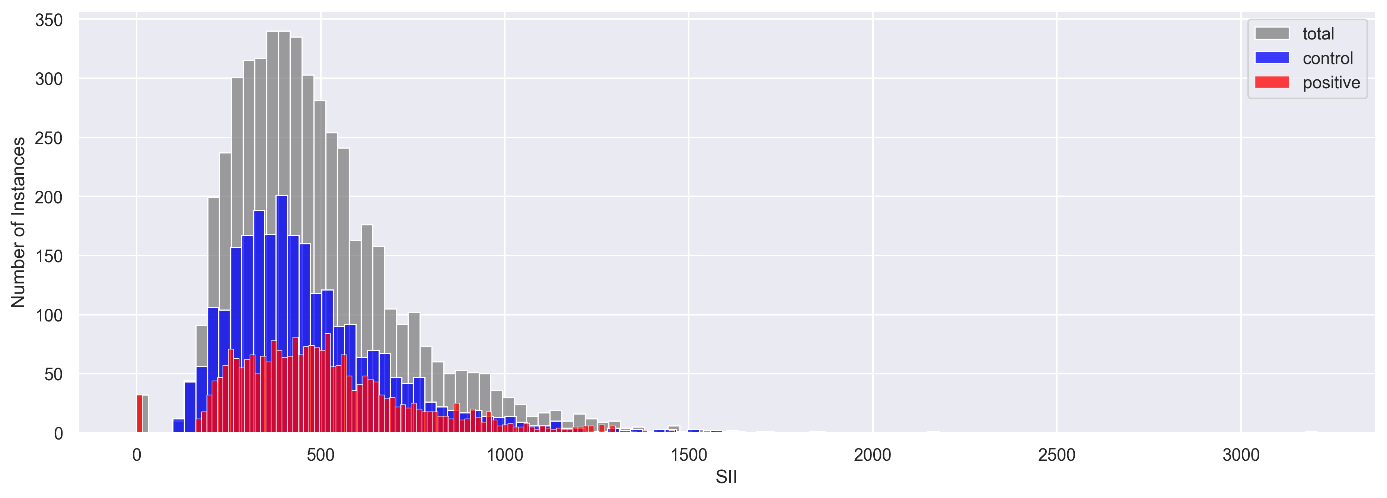
**

**o)**

**
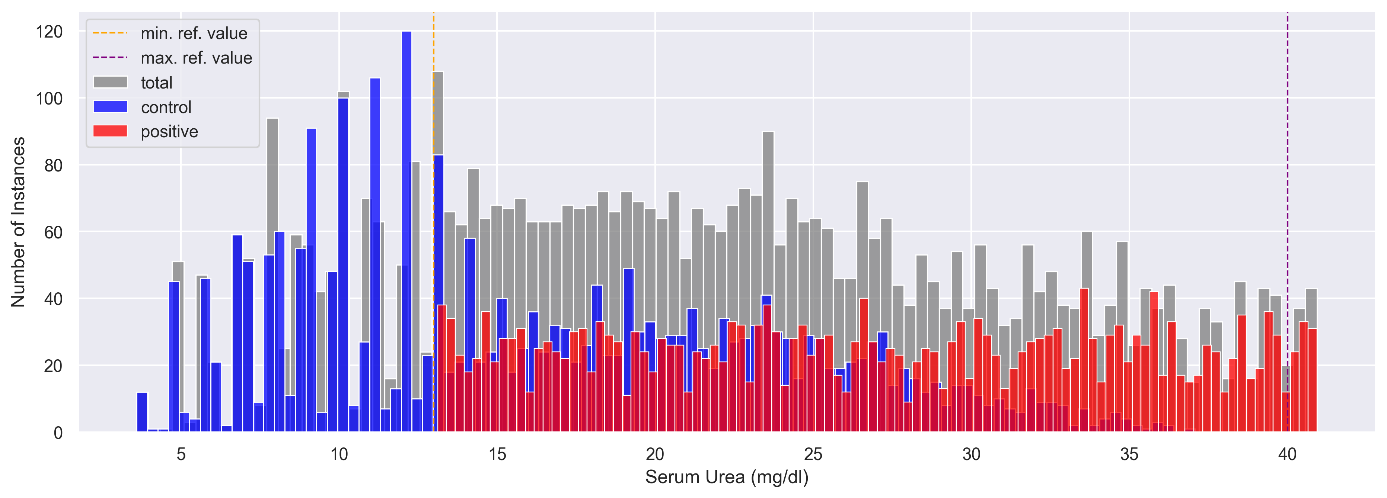
**

**p)**

**
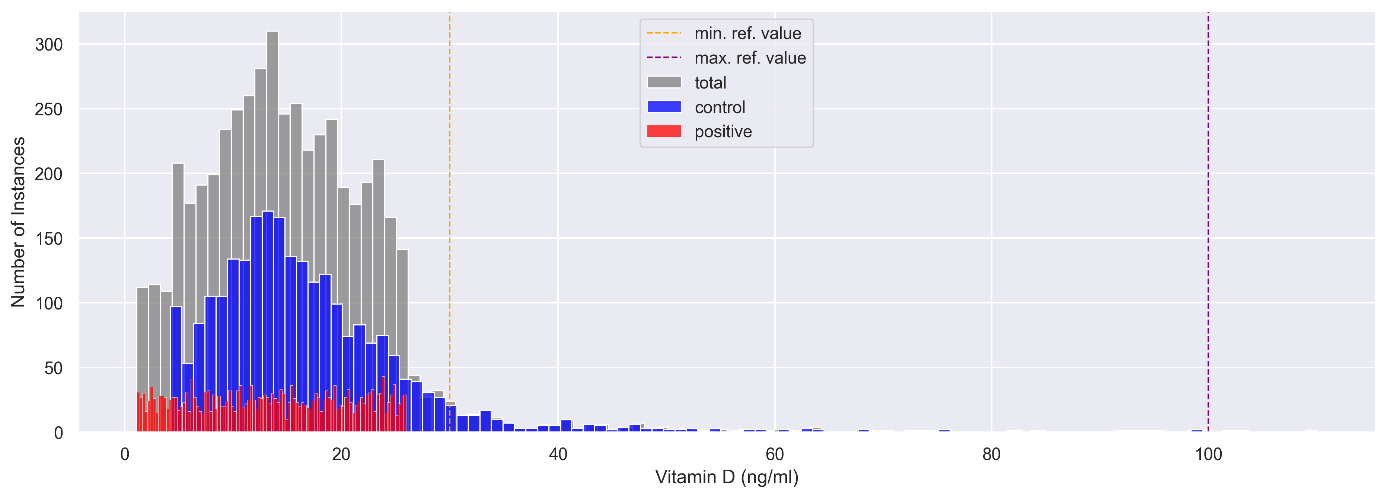
**

**Supplementary Figure 1.** Histograms presenting distribution of values for each parameter.

Footnote: X-axis represents a range of values of the given feature; Y-axis represents the number of data instances falling into the same bucket of values (values are grouped into 100 buckets). Blue colour denotes data from SARS-CoV-2 (-) (control group); red SARS-CoV-2 (+) (study group); grey the total number, i.e., control + positive.

a) Age b) Alanine transaminase c) Serum urea d) Serum cholesterol e) COVID f) Estimated glomerular filtration rate g) Gender h) Random serum glucose i) Lymphocytes j) Neutrophils k) Neutrophil-lymphocyte ratio l) Platelet count m) Red blood cells count n) Systemic immune-inflammation index o) Serum urea p) Vitamin D
